# Comparing views of patients and eye care professionals on the information provided on age-related macular degeneration and diabetic macular oedema

**DOI:** 10.1038/s41433-024-02939-z

**Published:** 2024-01-25

**Authors:** Sarah Clinton, Geraldine Hoad, Peter Bloomfield, Emma Malcolm, Karen Searle, Sarah Jarman, Rebecca Barber, Sarah Tucker

**Affiliations:** 1 Macular Society, Andover, UK; 2 Yellow Insights, Knutsford, UK

**Keywords:** Education, Quality of life

Both the National Institute for Health and Care Excellence (NICE) and the Royal College of Ophthalmologists Guidance for age-related macular degeneration (AMD) recommend that patients be provided sufficient information about their condition and the available support services [1, 2]. One of the established support service providers for these patients is the Macular Society. The aim of this study by the Macular Society was to evaluate the communication of necessary information between eye care professionals (ECPs) and patients.

## Methodology

Two 20-min online surveys were conducted with a multi-ethnic sample of relevant ECPs and patients, respectively. Screening questions, including awareness levels of the Macular Society for patients, determined the eligibility of participants. Each survey was composed of both structured and open-text questions. The patient survey was tailored to be suitable for low vision. This research was conducted in line with British Healthcare Business Intelligence Association UK guidelines [3].

## Participants

A total of 122 ECPs participated in the survey, 53 (43.4%) from primary care and 69 (56.6%) from secondary care. Out of the 214 patients who completed the survey, 90 (42.1%) were diagnosed with dry AMD, 81 (37.9%) with wet AMD and 43 (20.1%) had diabetic macular oedema (DMO). The majority (78%) of the patients had low awareness of the Macular Society.

## Results

**Fig. 1:**
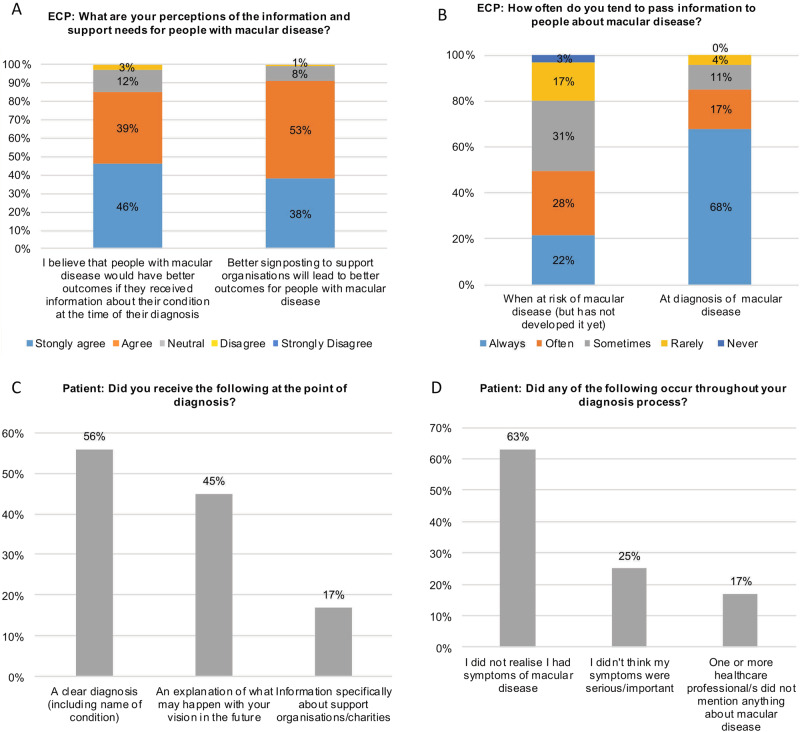
Survey results from eye care professionals and patients. **A** Eye care professionals were asked their views on the support needs for patients and **B** how often they provide patient information (n = 122). **C** Patients were surveyed on their experience at diagnosis receiving information and **D** understanding their condition (n = 214).

Figure 1 shows ECPs’ and patients’ responses to two questions each.

## Discussion

One key outcome of this study was the disparity between patient recall of information provision compared with ECPs signposting perceptions and stated behaviours. While ECPs agree that it is important to provide information about a patient’s condition and the available support services (Fig. 1A), many patients do not remember receiving this at diagnosis (Fig. 1C). A recent study looking at mental health and support services for those diagnosed with eye disease similarly found a lack of signposting for patients [4]. There could be many factors involved, including patients forgetting, or not being able to take in all the information provided.

While the majority of ECPs reported that they provide information about macular disease at diagnosis, only 21% of ECPs consistently provide information about macular disease to at risk patients (Fig. 1B). This can lead to late diagnosis because patients are not aware of the symptoms of macular disease and are not informed of the importance of regular eye checks. This was evident with 63% of patients not knowing that their symptoms were due to macular disease, and 25% not thinking these symptoms were important (Fig. 1D). For those with wet AMD and DMO where treatments are available, delays have been shown to lead to worse visual outcomes [5].

There may be barriers faced by ECPs in relaying the information effectively including time restraints, or fear of overburdening patients with information. On the other hand, patients may also experience challenges such as feeling overwhelmed, especially at diagnosis, difficulty reading leaflets, or accessing information.

This research highlights a clear need to optimise ECP communication around diagnosis, as well as more effective signposting of services provided by patient support organisations.

## Data Availability

The datasets generated during and analysed during the current study are available from the corresponding author upon reasonable request.

## References

[1] Royal College of Ophthalmologists Commissioning Guidance for AMD. 2021. https://www.rcophth.ac.uk/wp-content/uploads/2021/08/AMD-Commissioning-Guidance-Executive-Summary-June-2021.pdf.

[2] Age-related macular degeneration NICE guideline [NG82]. 2018. https://www.nice.org.uk/guidance/ng82.

[3] BHBIA Legal and Ethical Guidelines for Healthcare Market Research. 2023. https://www.bhbia.org.uk/guidelines-and-legislation/legal-and-ethical-guidelines.

[4] Trott M, Driscoll R, Bourne R, Slade J, Ingleton H, Farrell S (2023). Mental health support across the sight loss pathway: a qualitative exploration of eye care patients, optometrists, and ECLOs. Eye.

[5] Lim JH, Wickremasinghe SS, Xie J, Chauhan DS, Baird PN, Robman LD (2012). Delay to treatment and visual outcomes in patients treated with anti-vascular endothelial growth factor for age-related macular degeneration. Am J Ophthalmol.

